# Simulation Through Virtual Reality for the Management of Anxiety and Neuropathic Pain: Protocol for a Randomized Clinical Study

**DOI:** 10.2196/64781

**Published:** 2025-08-06

**Authors:** Jorge Muriel-Fernández, Jaime Gonçalves-Sánchez, Clemente Muriel-Villoria, Jesús María Gonçalves-Estella, José Manuel Lopez-Millán

**Affiliations:** 1 Department of Surgery, Universidad de Salamanca, Campus Miguel de Unamuno, Salamanca, 37007, Spain, +34 619045000; 2 Department of Surgery, Universidad de Sevilla, Sevilla, Spain

**Keywords:** neuropathic pain, virtual reality, pain management, anxiety, randomized controlled trial, non-pharmacological intervention, neuropathic, chronic pain, disability, quality of life, innovative, alleviate pain, distraction, efficacy, pain reduction

## Abstract

**Background:**

Neuropathic pain is a complex chronic pain condition often accompanied by affective symptoms such as anxiety and depression. This comorbidity is associated with increased pain severity, disability, and diminished quality of life, complicating treatment. Virtual reality (VR) is an innovative non-pharmacological intervention that is gaining attention for its potential to alleviate pain and anxiety through immersive distraction. The Protocol Committee of the University of Salamanca (Spain) has approved the study protocol (approval code: ID.1243)

**Objective:**

We aimed to evaluate the efficacy of VR in reducing pain and anxiety in patients with persistent neuropathic pain.

**Methods:**

This randomized, controlled, multicenter, open-label trial involves two groups: an intervention group receiving VR sessions and a control group receiving standard pharmacological treatment. Participants are adults aged 30‐61 years diagnosed with neuropathic pain, unresponsive to flexible doses of gabapentin. Pain and anxiety levels are assessed using the Pain Detect Questionnaire, Visual Analog Scale (VAS), and Goldberg Anxiety Scale at baseline and follow-up points. VR sessions are conducted weekly for 3 weeks, each lasting 30‐35 minutes. Perceived time during VR sessions is recorded as an indirect measure of distraction effectiveness. The primary outcome is the reduction in pain intensity and anxiety levels post-intervention compared to baseline. A sample size of 30 patients (15 per group) was calculated to achieve 80% statistical power, considering a 2-point mean difference in VAS scores.

**Results:**

A preliminary pilot study was completed with 16 patients, including 9 who received the virtual reality intervention. Initial findings showed reduced pain perception in 3 patients and decreased anxiety in 4 patients. These results informed the current protocol, which is scheduled to begin in 2025 and is currently in the preparatory phase.

**Conclusions:**

This study hypothesizes that VR will significantly reduce pain and anxiety in patients with persistent neuropathic pain. By providing new insights into non-pharmacological pain management strategies, this research aims to enhance the quality of life for these patients. The findings could support the broader application of VR in clinical pain management.

## Introduction

Pain is defined as a complex and multidimensional sensory experience that includes cognitive, behavioral, and psychological elements, often associated with unpleasant and subjective experiences. It has an adaptive function that initiates protective responses. Neuropathic pain is a form of chronic pain resulting from injury to the peripheral or central nervous systems. It is characterized as pain directly caused by an injury or disease affecting the somatosensory system [1].

Neuropathic pain frequently coexists with affective symptoms and negative emotions (anxiety, depression, fear, etc). It is estimated that 20%‐30% of patients with chronic pain also experience anxiety-depressive symptoms. Specifically, 50% of patients with chronic pain experience major anxiety-depression at some point, with 34% in the case of neuropathic pain [2,3].

The comorbidity of chronic pain and affective disorders is closely associated with higher pain severity, greater disability, and a substantial decline in patient quality of life [4], complicating treatment significantly. Persistent long-term pain and associated suffering seem to be key factors in developing secondary affective and emotional disorders. Long-term neuropathic pain has been shown to hyperactivate the LC-BLA pathway (that increases the activity of projections from the locus coeruleus [LC] to the basolateral amygdala [BLA]—a key noradrenergic circuit involved in arousal, emotional memory, and stress-related behaviors), responsible for anxiety symptoms secondary to the pain process [5].

Treating neuropathic pain is challenging. Pain management programs, therapeutic education, and physical training are fundamental for improving this type of pain. Therefore, it is crucial for patients to understand their pain process to change erroneous beliefs about it, as difficult-to-treat neuropathic pain is related to various psychosocial factors such as high stress or anxiety levels and various emotions caused by the pain.

Techniques aimed at reducing pain and anxiety are being introduced. One non-pharmacological, safe, and innovative technique that could potentially reduce pain and anxiety and is gaining interest in the scientific community in the “new technologies era” is virtual reality (VR). VR findings have helped to better understand the underlying processes of pain experiences and the analgesic role such strategies can play [6].

VR provides a three-dimensional, multisensory, and immersive environment that allows individuals to have modified reality experiences by creating a sense of “presence,” making it an excellent candidate for distraction-based therapy. This is suggested to decrease attention to painful stimuli, which could affect the emotional interpretation associated with it, thus reducing perceived intensity.

Distractions help reduce anxiety by preventing painful stimuli from being transmitted to the thalamus (ie, the limbic system) or the sensory cortex as effectively, helping to focus attention on external and internal stimuli rather than nociceptive stimuli. Sometimes, distractions surpass the capability of local anesthetics to control pain and discomfort associated with medical interventions. Distractions can be active (immersive), involving patient participation by manipulating the environment, or passive (non-immersive), involving only observation [7].

Current technological advances, especially in the field of VR, have resulted in new types of distractions that can be used alongside traditional distractions to achieve better pain control in patients.

In VR technology, users interact with a computer-simulated three-dimensional environment. VR provides multisensory information (visual, auditory, tactile, and olfactory stimuli) that helps individuals interact in the simulated world. A literature review by Lopez-Valverde et al [8] concluded that VR has shown its utility in reducing chronic pain and related issues such as anxiety, hopelessness, time spent thinking about pain, and perceived time during a procedure.

The concept of “virtual reality” lacks a unique and homogeneous definition among authors who have worked on it. We believe the most appropriate definition is by Riva [9], who conceptualized VR as a communication interface based on interactive three-dimensional visualization capable of collecting and integrating different datasets into a single realistic experience. Various theories have been proposed regarding pain-modulating effects to explain VR’s effects. To understand VR-induced hypoalgesia mechanisms, it is necessary to integrate neurobiological and neurochemical interactions with cognitive-attentional and affective-emotional aspects of the “painful experience” [10].

One of the main hypotheses states that humans have a limited capacity to pay attention to stimuli, so experiencing pain requires some attention to it [11]. Additionally, patients with both acute and chronic pain may experience excessive attention and vigilance towards pain, potentially increasing perceived intensity (or sensitivity to painful stimuli). VR could have an analgesic effect by distracting from the pain focus [12], producing a pain modulation response via neurophysiological activity in brain areas related to analgesia.

However, distraction is not the only proposed effect. The neuromatrix theory suggests that the pain experience results from a complex interaction between attentional, neurobiochemical, and emotional factors specific to each person. Therefore, pain depends on the interpretation and response of the brain matrix. In this context, VR could promote intercortical analgesia by modulating attentional and emotional networks, stimulating areas related to pain inhibition.

Many aspects remain unclear; however, VR use for pain treatment has shown significant benefits.

This study protocol aims to evaluate the outcomes in terms of pain and anxiety relief with VR application in this group of patients with persistent neuropathic pain.

## Methods

### Ethical Considerations

This study will adhere to the principles outlined in the Declaration of Helsinki and has been approved by the Institutional Review Board of the participating centers (University of Salamanca: approval code: ID.1243). Written informed consent (Multimedia Appendix 1) will be obtained from all participants before study enrollment.

### Study Design

#### Trial Design

We will conduct a randomized, controlled, multicenter, open-label, parallel-group trial with two assigned groups (intervention group and control group).

#### Scope and Study Period

**Figure 1.**
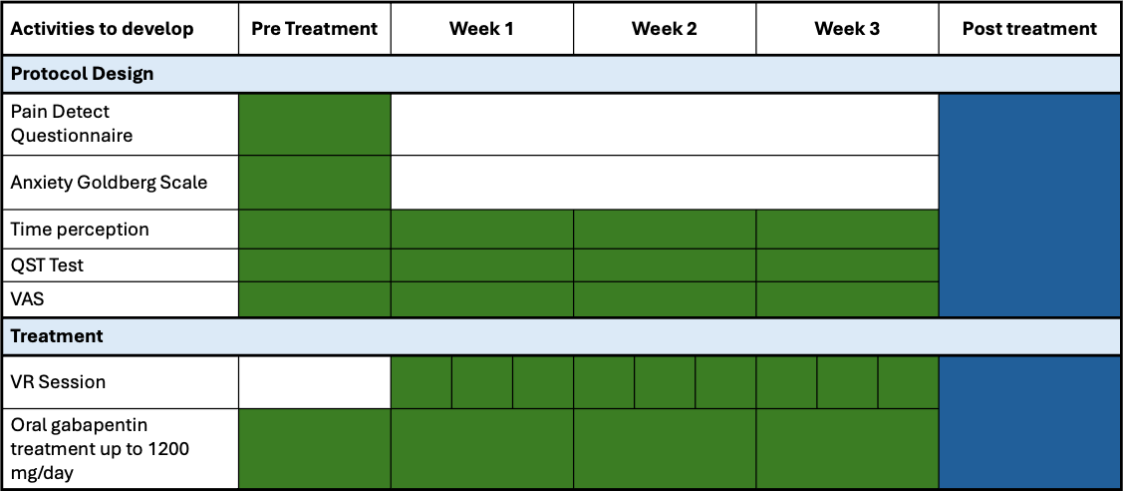
Temporal planning of the activity development during the project. VAS: visual analog scale; VR: virtual reality.

The study population will include adults aged 30 to 61 years. The study was started by the beginning of 2025, as shown in Figure 1.

### Participants

All patients will be evaluated and deemed suitable by a specialist in the Pain Unit. Neuropathic pain diagnosis was made using the Pain Detect Questionnaire [13].

Neuropathic pain symptoms and signs will be assessed, including sensitivity alterations history such as tingling, numbness, stabbing, burning, shooting, or electric-like pain, paresthesia, allodynia, hyperalgesia, hyperpathia, and motor dysfunction (foot or wrist drop, symmetric motor weakness, difficulty buttoning a shirt or holding a pencil), myalgia, or muscle cramps. Neurological assessment included motor evaluation (muscle strength in limbs), sensory evaluation (touch, pain [pinprick test], cold, heat, vibration, and position, and allodynia test for dynamic and static mechanical stimuli), balance test, coordination, and deep tendon reflexes.

After diagnosing persistent neuropathic pain, oral gabapentin treatment will be prescribed up to 1200 mg/day.

### Inclusion Criteria

Patients will be included if they have moderate to severe pain intensity despite treatment with flexible doses of gabapentin for 3 months up to 1200 mg/24 h, understand VR operation and use of validated scales, and sign informed consent forms (Informed consent available as appendix).

### Exclusion Criteria

The exclusion criteria include known hypersensitivity to gabapentin, inability to tolerate 1200 mg/24 h doses (dizziness and drowsiness), pregnant or lactating women, any relative or absolute contraindication to VR treatment, patients with unstable or poorly controlled hypertension, recent cardiovascular events (6 mo) before starting treatment, long-term diabetics with cardiovascular autonomic neuropathy, elderly patients, or those with cognitive deficits who cannot understand or apply the scales or answer the questions correctly.

### Randomization

#### Sequence Generation

Randomization will be performed using the RandomizedR computer system (CRAN-R Project), a randomization tool in the R Studio program.

#### Implementation

Participants will be selected from a daily patient registry. Both the sequence and participant assignment to interventions will be generated by the RandomizedR computer system.

#### Masking

Due to the study’s nature, it will not be possible to blind patients or health care professionals. Therefore, the trial will be open-label. However, a third-party blind evaluation will be conducted, as the data analysis responsible will not be involved in the intervention.

### Data Collection, Sources of Information, and Intervention

Pain intensity will be measured using the PainDETECT Questionnaire [13], which is self-administered with 9 descriptors. It detects neuropathic pain intensity and discriminates between possible neuropathic pain (scores between 12 and 19) and definite neuropathic pain (scores above 19). The Visual Analog Scale (VAS) is a one-dimensional quantitative, effective, and easily reproducible scale. Patients mark a 100-mm segment with endpoints labeled as no pain (0 mm) and maximum pain (100 mm); the measure from 0 to the patient-marked point indicates pain intensity. A change of 20 mm (or 2 points on a 10-point pain intensity scale) is considered clinically significant.

Anxiety will be measured using the Goldberg Anxiety Scale [14], a test that not only guides diagnosis but also measures intensity. It contains 2 subscales with 9 questions each: anxiety subscale (questions 1‐9). The first 4 questions of each subscale (questions 1‐4) act as a precondition to determine if the rest of the questions should be answered. Specifically, if fewer than 2 questions of the first 4 are answered affirmatively, the rest of the first subscale questions should not be answered. For the second subscale, it is sufficient to answer one of the questions 10‐13 affirmatively to proceed with the rest of the questions.

Time perception will be analyzed as an indirect measure of distraction, with less attention to pain, greater tolerance, and possibly an early sensation of completing the treatment time (or Tprogrammed).

At the end of each VR session (30 to 35 minutes), patients will be asked how much time they think remains. This is called “perceived time” (Tperceived) and will be recorded in 10‐15-minute intervals based on their responses.

A “significant” difference is defined as a time difference (Tprogrammed - Tperceived) of ≥15 min and “no change” if ≤10 min. For patients who request to stop before completing the time, their “perceived time” is noted by adding the remaining time to the total programmed time.

The frequency and duration of the intervention—fifteen 30-minute VR sessions over 3 consecutive weeks (Monday to Friday)—were selected based on previous studies demonstrating that repeated short-duration sessions enhance user immersion and treatment adherence without inducing fatigue or diminishing attention [15]. This schedule allows for progressive desensitization to pain stimuli and anxiety reduction through consistent exposure to immersive distraction.

For the VR application, the Oculus Go VR 32GB (Meta) model was used, featuring a head-mounted display. This headset provides a stereo visual image, thereby creating a sense of space and depth. A motion tracker in the headset’s screen measures head position and adjusts the visual image accordingly. As a result, users perceive their surroundings and can move through the simulated environment. The headset is accompanied by headphones that provide sounds, further enhancing the user’s immersion in the virtual world.

Participants in the control group underwent the usual pain assessment review during the 2-week study period.

Eligible patients will be invited to participate in the study continuously, and the assignment to study groups will be randomized.

### Sample Size

To detect a difference of 1 point between the two groups on the pain level scales, a sample of 50 patients is required, with 25 in each group, assuming an SD of 3 points, a 95% confidence level, a Z-score of 1.96, a margin of error *α*<.05, and an estimated 10% dropout rate [15].

### Patient Recruitment for Study Participation

#### Patient Selection

Patients included in the study will be selected from individuals diagnosed with persistent neuropathic pain by Pain Units, with moderate to severe intensity. All will be duly informed about the nature and objectives of the study.

Twenty-five patients will be included, and their scores on the VAS, the Pain Detect Questionnaire, and perceived time will be analyzed.

Participants will be adults aged 30 to 61 years. This age range was selected to reduce clinical variability and cognitive interference associated with younger or older populations. Individuals aged under 30 years may not reflect the target patient population for chronic neuropathic pain in clinical practice, while individuals aged over 61 years may present comorbidities or age-related cognitive decline that could interfere with VR engagement or outcome assessments.

#### Control Selection

Controls will be selected from patients in the Pain Unit diagnosed with moderate to severe neuropathic pain. All will be duly informed about the nature and objectives of the study and asked to provide signed consent.

Before the study begins, training on the use of the devices will be provided to all health care personnel involved.

#### Data Collection, Information Sources, and Intervention

Data collection will begin once the patient has provided written informed consent. Patient assignment to a study group will be randomized using the RandomizedR computer system.

The data collector will record the patients’ age, sex, study group, and type of intervention performed. Before contacting the patient with the virtual reality device, the patient’s status and heart rate will be recorded upon arrival at the clinic, regardless of the assigned study group.

Patients in Group 1 will be the intervention group. They will undergo 10 virtual reality pain treatment sessions (Monday to Friday for 2 weeks), with each session lasting 30 minutes, while maintaining treatment with gabapentin at a dose of 1200 mg/24 h. Their clinical history, pain situation, and anxiety will be assessed in each session.

Patients in Group 2 will be the control group. Their clinical history, pain situation, and anxiety will be assessed in each session. They will receive pain treatment with oral gabapentin at a dose of 1200 mg/24 h. The control group will receive standard care without any VR exposure. Participants will remain in a quiet room with standard analgesic treatment as prescribed. No additional relaxation stimuli will be provided. Outcome data will be collected in parallel with the intervention group.

**Figure 2.**
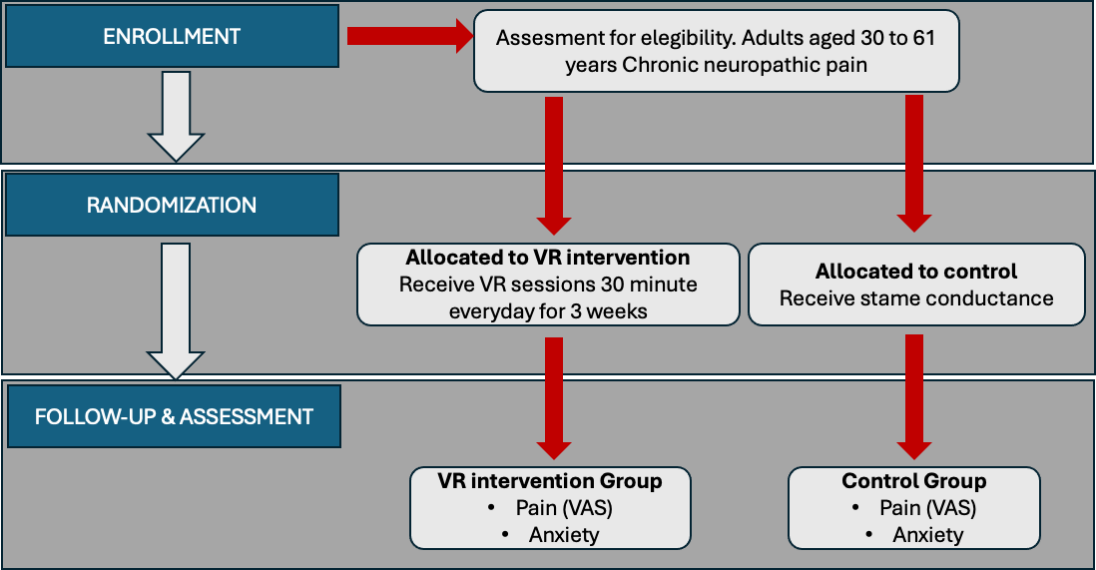
CONSORT diagram used in clinical trial reporting to track the flow of participants through a trial, showing how many were screened, eligible, randomized, and completed each stage of the study.

Figure 2 shows the CONSORT diagram.

### Data Analysis

Data entry for anxiety and pain test scores through VAS, Pain Project, and perceived time will be performed using Microsoft Excel and analyzed using SPSS statistical software (version 16.0).

A sample *t* test will be used. Before applying the *t* test, the Kolmogorov-Smirnov test will be used to evaluate the normality of the mean scores obtained.

Likert-type statements will be analyzed such that a score below 3 indicates disagreement and a score above 3 indicates agreement with a positive statement and vice versa for a negative statement.

Intervention group patients will be informed that they can use the VR device. They will be assisted in putting on the device and given a brief explanation of the content to be displayed.

Data collected from the control group will be compared with those from the intervention group (heart rate, pain perception level, anxiety level).

The mentioned data will be collected by an IT professional through a web questionnaire generated by Microsoft Forms on a tablet and will be stored on a computer server.

### Statistical Analysis

Subjects will be analyzed according to the group to which they were assigned.

Data will be collected through Microsoft Forms (an application included in Office 365 by Microsoft Corporation that allows for creating customized questionnaires, surveys, and records) and analyzed with R software (version 4.0.3). Categorical variables will be described using absolute frequencies and percentages, and continuous variables will be described using means and standard deviations or medians and quartiles. A two-tailed *t* test will be used to compare values related to pain, anxiety, and satisfaction between the two groups. Correlations between pain perception and anxiety values reported by patients will be assessed using Pearson’s correlation. The significance level will be set at 5%, and all confidence intervals will be set at 95%.

Data will be stored in a database. Pearson’s *χ*^2^ test will be used to calculate statistical significance.

## Results

The current study began in April 2025 and is currently in the preparatory phase. Previously, a preliminary pilot study was conducted in which 16 patients were recruited, of whom 9 received the VR intervention [16].

In this pilot study, using a distraction chosen by each participant, a significant reduction in pain (defined as a decrease of ≥2 points on the VAS compared to previous experience) was observed in 3 patients. Five patients reported no noticeable change (VAS difference of 0 or 1 points), and 1 patient experienced worsening symptoms and discontinued participation.

Additionally, anxiety, measured using the Goldberg Scale, decreased during the session in 4 patients, with “significant anxiety” defined as a score >2 points.

Regarding time perception, 50% of participants reported a notable difference in their perception of time passage 30 days after the intervention (considered significant if the treatment duration was perceived as 30 min).

These preliminary results provided the rationale for the current full-scale randomized controlled trial, which aims to evaluate whether the use of virtual reality is effective in reducing anxiety and chronic neuropathic pain. In the current protocol, a statistically significant reduction in heart rate and pain intensity in the intervention group compared to the control group will be considered a satisfactory outcome.

If the hypotheses are confirmed, this intervention could be implemented in routine care and extended to other procedures involving difficult-to-manage pain.

## Discussion

### Principal Findings

This protocol outlines a randomized controlled trial aimed at evaluating the impact of VR on reducing anxiety and chronic neuropathic pain. Based on a previous pilot study and literature supporting the use of immersive distraction therapies [6,8,10], we hypothesize that VR will significantly improve patient-reported outcomes in terms of pain perception, anxiety levels, and treatment satisfaction.

### Comparison With Prior Work

Several previous studies have demonstrated the analgesic and anxiolytic effects of VR in clinical contexts such as dental procedures, burn care, and pediatric interventions [7,8,17-19]. However, there is a scarcity of research targeting adults with pharmacoresistant neuropathic pain, a population with limited treatment options [20]. Our protocol addresses this gap and builds upon evidence showing that immersive experiences can activate cortical regions involved in pain modulation [12].

### Strengths and Limitations

A strength of this study is the integration of immersive VR with standard pharmacologic therapy in a structured and replicable design. Limitations include the lack of home-use devices, potential biases in self-reported outcomes, and the absence of personalization in VR content. Additionally, the exclusion of older adults limits generalizability.

### Future Directions and Dissemination

Future studies should explore the feasibility of home-based VR systems and allow patients to select content tailored to their preferences to maximize engagement. Results of this study will be disseminated through peer-reviewed journals, clinical conferences, and institutional briefings, supporting broader implementation in pain management strategies.

### Conclusion

If the expected outcomes are confirmed, VR could represent a scalable, non-pharmacological adjunct for managing chronic pain and anxiety, potentially transforming routine clinical practices.

## Supplementary material

10.2196/64781 Multimedia Appendix 1 Informed Consent.

## Abbreviations

VAS: visual analog scale
VR: virtual reality

## References

[1] Merksey H, Bogduk N, IASP Task Force in Taxonomy (1994). Classification of Chronic Pain Syndromes and Definitions of Pain Terms.

[2] McWilliams LA, Cox BJ, Enns MW (2003). Mood and anxiety disorders associated with chronic pain: an examination in a nationally representative sample. Pain.

[3] Bair MJ, Robinson RL, Katon W, Kroenke K (2003). Depression and pain comorbidity: a literature review. Arch Intern Med.

[4] Bair MJ, Wu J, Damush TM, Sutherland JM, Kroenke K (2008). Association of depression and anxiety alone and in combination with chronic musculoskeletal pain in primary care patients. Psychosom Med.

[5] Llorca-Torralba M, Suárez-Pereira I, Bravo L (2019). Chemogenetic silencing of the locus coeruleus-basolateral amygdala pathway abolishes pain-induced anxiety and enhanced aversive learning in rats. Biol Psychiatry.

[6] Li A, Montaño Z, Chen VJ, Gold JI (2011). Virtual reality and pain management: current trends and future directions. Pain Manag.

[7] Hoffman HG, Doctor JN, Patterson DR, Carrougher GJ, Furness TA (2000). Virtual reality as an adjunctive pain control during burn wound care in adolescent patients. Pain.

[8] López-Valverde N, Muriel Fernández J, López-Valverde A (2020). Use of virtual reality for the management of anxiety and pain in dental treatments: systematic review and meta-analysis. J Clin Med.

[9] Riva G (2005). Virtual reality in psychotherapy: review. Cyberpsychol Behav.

[10] Smith V, Warty RR, Sursas JA (2020). The effectiveness of virtual reality in managing acute pain and anxiety for medical inpatients: systematic review.. Nov 2, 2020. J Med Internet Res.

[11] Wismeijer AAJ, Vingerhoets A (2005). The use of virtual reality and audiovisual eyeglass systems as adjunct analgesic techniques: a review of the literature. Ann Behav Med.

[12] Hoffman HG, Richards TL, Van Oostrom T (2007). The analgesic effects of opioids and immersive virtual reality distraction: evidence from subjective and functional brain imaging assessments. Anesthesia & Analgesia.

[13] De Andrés J, Pérez-Cajaraville J, Lopez-Alarcón MD (2012). Cultural adaptation and validation of the painDETECT scale into Spanish. Clin J Pain.

[14] Goldberg DP, Hillier VF (1979). A scaled version of the general health questionnaire. Psychol Med.

[15] Garrett B, Taverner T, Masinde W, Gromala D, Shaw C, Negraeff M (2014). A rapid evidence assessment of immersive virtual reality as an adjunct therapy in acute pain management in clinical practice. Clin J Pain.

[16] J MF, C Muriel V, Mj SL, Jm GE, Jm LM (2023). Virtual reality in the treatment of anxiety and chronic neuropathic pain. Preliminary study. Ann Psychiatry Treatm.

[17] de la Cruz Herrera M, Fuster-Casanovas A, Miró Catalina Q (2022). Use of virtual reality in the reduction of pain after the administration of vaccines among children in primary care centers: protocol for a randomized clinical trial. JMIR Res Protoc.

[18] Jeffs D, Dorman D, Brown S (2014). Effect of virtual reality on adolescent pain during burn wound care. J Burn Care Res.

[19] Gupta A, Scott K, Dukewich M (2018). Innovative technology using virtual reality in the treatment of pain: does it reduce pain via distraction, or is there more to it?. Pain Med.

[20] Overton M, Swain N, Falling C, Gwynne-Jones D, Fillingim R, Mani R (2024). Activity-related pain and sensitization predict within- and between-person pain experience in people with knee osteoarthritis: An ecological momentary assessment study. Osteoarthr Cartil Open.

