# Supplementary material for: Simulation Through Virtual Reality for the Management of Anxiety and Neuropathic Pain: Protocol for a Randomized Clinical Study
Source: JMIR Res Protoc. 2025 Aug 6;14:e64781. doi: 10.2196/64781 (PMC12360831; doi:10.2196/64781)
Supplement: Multimedia Appendix 1 [file resprot-v14-e64781-s001.docx]

**Impreso CEI-A1**

**HOJA DE INFORMACIÓN AL PARTICIPANTE Y CONSENTIMIENTO INFORMADO PARA PROYECTOS DE INVESTIGACIÓN EN PERSONAS CON PLENA CAPACIDAD**

**Título:** **La Simulación a través de la realidad virtual en el manejo del dolor neuropático persistente: Protocolo para un estudio clínico controlado aleatorizado, multicéntrico, abierto, paralelo**

**I.P.: Jorge Muriel Fernández**

**Introducción**

Se le ha invitado a participar en un estudio de investigación. Por favor, tómese el tiempo que necesite para leer la siguiente información y consultar lo que desee. Pregúntele al/la investigador/a de este estudio si hay algo que no le queda claro o si desea obtener más información.

Este proyecto ha sido informado favorablemente por el Comité de Ética de la Investigación de la Universidad de Salamanca y sigue las recomendaciones éticas de la declaración de Helsinki.

Nuestra intención es que reciba la información correcta y suficiente para que pueda evaluar y juzgar si quiere o no participar en este estudio. Para ello lea esta hoja informativa con atención y nosotros le aclararemos las dudas que le puedan surgir. Además, puede consultar con las personas que considere oportuno.

**Objetivo del estudio**

Considerando los resultados del tratamiento farmacológico que Usted tiene y que no le permite la solución del dolor en su totalidad se pretende complementar el estudio con sesiones de Simulación con realidad virtual.

Los resultados de este estudio en el supuesto que sean positivos pueden proveer de poderosas herramientas de intervención para mejorar las habilidades para el tratamiento más correcto de los pacientes en el dolor neuropático persistente.

**Procedimientos**

Se incluirán 40 pacientes

Se realizarán en dos grupos uno con tratamiento farmacológico más realidad virtual y otro solo con tratamiento farmacológico, serán asignados los pacientes de forma aleatoria utilizando un sistema informático.

Se debe decir que “ni el médico ni el paciente sabrán cuál es el tratamiento que va a recibir”, la valoración de los resultados se realizara por una tercera persona.

Se realizan 10 sesiones de tratamiento del dolor mediante tratamiento farmacológico y realidad virtual (de lunes a viernes durante dos semanas), de 30 minutos por sesión diaria de duración cada una.

**Principio de no maleficencia: Riesgos y molestias**

La participación en este estudio no produce ninguna molestia, y no implica riesgo alguno para la salud

No existen riesgos y molestias de las pruebas que se realizan como consecuencia del estudio.

Usted tiene la responsabilidad de participar en el estudio por lo que debe:

- Comprometerse a realizar al Hospital las visitas y actividades del estudio

- Notificar cualquier evento adverso que le suceda o cambios en medicación.

**Posibles beneficios**

No se espera un beneficio directo por su participación en el estudio. No obstante, los conocimientos obtenidos gracias a estudio pueden ayudar al avance médico y, por ello, a otras personas.

**Cesión de datos o muestras**

En caso de que sus muestras y/o sus datos sean cedidos a otros grupos de investigación, se realizará siempre según la legislación vigente, con sus datos codificados, y para realizar exclusivamente estudios relacionados con los objetivos de este trabajo, y con previa autorización del Comité de Ética de la Investigación de la Facultad de Medicina de la Universidad de Salamanca*.* En caso de que los objetivos del trabajo de investigación propuesto por otros grupos de investigación sean diferentes a los del presente proyecto, se le solicitará un nuevo consentimiento.

No percibirá ningún beneficio económico por su participación en este estudio.

**Principio de autonomía y beneficios de su participación: Participación y retirada voluntarias**

Usted puede decidir libremente si desea o no tomar parte en este estudio, la participación es totalmente voluntaria. Si decide participar, sigue teniendo la posibilidad de retirarse en cualquier momento y sin tener que dar explicaciones, y sin penalización alguna ni consecuencias negativas para Ud. Su decisión de retirarse no le afectará para nada. Si decide participar, debe comprometerse a realizar lo mejor posible lo que le indique el equipo investigador.

**Derecho a la información**

No se espera un beneficio directo por su participación en el estudio. No obstante, los conocimientos obtenidos gracias a estudio pueden ayudar al avance médico y, por ello, a otras personas.

Sí deseo recibir los resultados generales de la investigación.

No deseo recibir información.

**Confidencialidad y medidas de seguridad**

Toda la información utilizada durante este estudio se tratará de manera estrictamente confidencial de acuerdo con la política de privacidad (ver hoja adjunta).

Se ha establecido un sistema de anonimización efectivo que no permite la identificación posterior de los participantes. En ningún caso se juntarán los consentimientos otorgados, donde sí se identifica a los participantes, con los cuestionarios o el resto de información utilizada en el estudio. En el uso que se realice de los resultados del estudio, con fines de docencia, investigación y/o publicación, se respetará siempre la debida anonimización de los datos de carácter personal, de modo que los participantes de la investigación no resultarán identificados o identificables.

Si los resultados del estudio fueran susceptibles de publicación en revistas científicas, en ningún momento se proporcionarán datos personales de los/las participantes en esta investigación.

Es importante que no comente las características de los procedimientos o los objetivos de este estudio hasta que haya concluido toda la investigación.

**Datos de contacto del equipo investigador:**

Nombre: ........................................................

Teléfono: ........................................................

Nombre: ........................................................

Teléfono: ........................................................

Nombre: ........................................................

Teléfono: ........................................................

**CONSENTIMIENTO INFORMADO PARA PERSONAS CON PLENA CAPACIDAD**

**Título:**

Yo *(Nombre, Apellidos y DNI) ________________________________*

He podido hacer preguntas sobre el estudio.

He hablado con el/la Investigador/a *______________________*

He recibido suficiente información sobre el estudio.

He leído la hoja de información que se me ha entregado.

Estoy informado del modo en que serán tratados mis datos.

Comprendo que mi participación es voluntaria.

Comprendo que puedo retirarme del estudio:

1º Cuando quiera.

2º Sin tener que dar explicaciones.

3º Sin que tenga ninguna repercusión negativa.

Acepto voluntariamente participar en el Proyecto y autorizo el uso de toda la información obtenida. Entiendo que recibiré una copia firmada de este consentimiento informado.

Firma del/la participante Fecha

Nombre y firma del/la investigador/a Fecha

**REVOCACIÓN DEL CONSENTIMIENTO**

Revoco el consentimiento prestado en fecha _____________ para participar en el proyecto titulado “_____________________________________________________” y, para que así conste, firmo la presente revocación.

En _____________________ , a ________ de _________________________ de 20___.

Firma del/la participante Fecha

Nombre y firma del/la investigador/a Fecha

**POLÍTICA DE PRIVACIDAD**

**¿Quién trata sus datos?**

El responsable del tratamiento de sus datos es:

Universidad de Salamanca

C.I.F. Q3718001E

C/ Patio de las Escuelas Menores, nº 1

C.P. 37008, Salamanca

**¿Cómo puede contactar con nuestro delegado de protección de datos?**

El delegado de protección de datos es la persona encargada de supervisar que cumplimos las normas sobre protección de datos y ayudarte. Si tienes alguna duda o consulta sobre cómo tratamos los datos puedes contactar con el delegado de protección de datos en: [dpd@usal.es](mailto:dpd@usal.es)

**¿Para qué tratamos sus datos? ¿Por qué y con qué base legal tratamos tus datos?**

Trataremos sus datos con el fin de gestionar su participación en el Proyecto de Investigación. Sus datos serán tratados en virtud de:

Su consentimiento (artículo 6.1.a) RGPD), para participar en el Proyecto, y la publicación de los resultados, en su caso, con relación a las referencias biográficas cuya publicación pudiera ser necesaria en el Proyecto.

Cumplimiento de una misión realizada en interés público o en el ejercicio de poderes públicos conferidos al responsable del tratamiento (art. 6.1.e) RGPD) conforme a las competencias atribuidas a la Universidad en virtud de los artículos 1 y 39 y siguientes de Ley Orgánica 6/2001, de 21 de diciembre, de Universidades.

**¿Con quién compartimos sus datos?**

▪ Únicamente se comunicarán los datos sin necesidad de otorgar consentimiento a requerimiento de autoridades.

En estos casos, la Universidad antes de poner los datos a disposición de terceros se asegura de que estas autoridades solicitan y acceden a los datos de acuerdo con las Leyes.

**¿Cuánto tiempo conservaremos los datos?**

▪ Los datos se utilizarán durante toda la investigación hasta, en su caso, la emisión de un informe o la publicación de los resultados de la misma.

▪ La información se conservará debidamente bloqueada por los periodos adicionales necesarios para la prescripción de eventuales responsabilidades legales.

▪ La información con valor histórico se conservará de forma indefinida previa aprobación de la Comisión de Expurgo en virtud de lo regulado en la Ley 16/1985, de 25 de junio, del Patrimonio Histórico Español y la normativa específica aplicable en su caso

**¿Cómo protegemos la información?**

Como Administración pública, aplicamos las medidas técnicas y organizativas que nos dicta el Esquema Nacional de Seguridad. Este contempla una serie de recomendaciones para tratar de garantizar la seguridad de los sistemas de información y así evitar el robo, alteración o accesos no autorizados a datos.

En caso de subcontratación de servicios, exigiremos y velaremos para que el encargado del tratamiento aplique medidas análogas a las del Esquema Nacional de Seguridad.

**¿Qué derechos tiene?**

Para poder mantener en todo momento el control sobre sus datos tienes derecho a acceder a su información personal, así como a solicitar la rectificación de los datos inexactos o, en su caso, solicitar su cancelación o supresión. En determinadas circunstancias, y por motivos relacionados con su situación particular, podrá oponerse al tratamiento de sus datos. De igual forma, puede ejercer el derecho de limitación del tratamiento de su información personal, solicitándonos su conservación y también la portabilidad de sus datos.

El ejercicio de derechos es personal y por ello necesitamos identificarle de modo inequívoco. Puedes ejercer tus derechos de dos modos:

▪ Mediante el envío de un mensaje de correo electrónico.

Para ello, utilice esta dirección: [dpd@usal.es](mailto:dpd@usal.es). Únicamente atenderemos las solicitudes que se realicen desde cuentas de correo electrónicos proporcionadas por la Universidad de Salamanca o que consten en nuestras bases de datos previa identificación de su titular.

▪ Mediante la presentación de un escrito en nuestro Registro o por correo postal dirigido a:

Secretaría General

Universidad de Salamanca.

C.I.F. Q3718001E

C/ Patio de las Escuelas Menores, nº 1

C.P. 37008, Salamanca

Debes aportar la siguiente documentación acreditativa:

▪ Acreditación de la identidad del interesado mediante cualquier documento válido, como DNI o pasaporte.

▪ Nombre y apellidos del interesado o, cuando corresponda, de la persona que le represente, así como el documento acreditativo de tal representación.

▪ Petición en que se concreta la solicitud.

▪ Dirección a efectos de notificaciones, fecha y firma del solicitante.

▪ Documentos acreditativos de la petición que formulas, si corresponde.

▪ En caso de la rectificación o cancelación, indicación del dato a rectificar o cancelar y la causa que lo justifica.

**¿Quién garantiza sus derechos? ¿Ante quién puede reclamar?**

En caso de que desee presentar una reclamación u obtener información adicional sobre la regulación del tratamiento de datos personales en España, la autoridad competente es la Agencia Española de Protección de Datos (Jorge Juan, 6 28001-Madrid).

**CONSENTIMIENTO PARA EL TRATAMIENTO DE DATOS Y CESIÓN DE DERECHOS DE IMAGEN PARA LA INVESTIGACIÓN**

D./Doña _____________________________________________, con DNI ____________, con pleno conocimiento y facultades, autorizo:

El tratamiento de los datos para los fines de la investigación descrita en el documento de Consentimiento Informado adjunto a la presente autorización.

La fijación, grabación y uso de imágenes y audio.

**a.- Fines generales del tratamiento de datos.**

Trataremos sus datos con el fin de gestionar su participación en el Proyecto de Investigación titulado ___________________________________. Sus datos serán tratados en virtud de:

- Su consentimiento (artículo 6.1.a) RGPD), para participar en el Proyecto, para el tratamiento de su imagen y la publicación de los resultados, en su caso, con relación a las referencias biográficas cuya publicación pudiera ser necesaria en el Proyecto.
- Cumplimiento de una misión realizada en interés público o en el ejercicio de poderes públicos conferidos al responsable del tratamiento (art. 6.1.e) RGPD) conforme a las competencias atribuidas a la Universidad en virtud de los artículos 1 y 39 y siguientes de Ley Orgánica 6/2001, de 21 de diciembre, de Universidades.

**b.- Registros de imagen o sonido.**

En el marco del desarrollo de la investigación se obtendrán fotografías, o registros de audio o vídeo. Ud. Autoriza a la Universidad de Salamanca al uso, edición, difusión y explotación de las imágenes exclusivamente para fines docentes y de investigación. En caso de utilización, se asegurará que el afectado nunca sea identificado por su nombre ni mediante información alguna que le haga identificable, salvo que conste consentimiento expreso y específico al efecto.

Todo ello con la única salvedad y limitación de aquellas utilizaciones o aplicaciones que pudieran atentar a los derechos garantizados en la Ley Orgánica 1/1982, de 5 de mayo, de Protección Civil al Derecho al Honor, la Intimidad Personal y familiar y a la Propia Imagen, así como del pleno respeto de las previsiones específicas del art. 4 de la Ley Orgánica 1/1996, de 15 de enero, de protección jurídica del menor.

**c.- Otra información relevante para la garantía de derechos en materia de protección de datos:**

**¿Quién es el responsable del tratamiento?**

El responsable del tratamiento de sus datos es:

Universidad de Salamanca

CIF: Q-3718001-E

C/ Patio de las Escuelas Menores, nº 1

C.P. 37008, Salamanca.

**¿Cómo obtenemos la información personal?**

Mediante la formalización de este impreso de consentimiento.

**¿A quiénes comunicamos o cedemos los datos? Destinatarios de la información.**

Los datos no serán comunicados ni cedidos a ningún tercero, salvo que los mismos sean exigibles por los Jueces y tribunales u otra autoridad pública en el ejercicio de sus funciones y de acuerdo con lo dispuesto en la normativa específica aplicable en su caso.

**¿Durante cuánto tiempo conservamos los datos?**

Los datos personales proporcionados se conservarán durante el periodo de desarrollo del proyecto de investigación.

La información se conservará debidamente bloqueada por los periodos adicionales necesarios para la prescripción de eventuales responsabilidades legales.

La información con valor histórico se conservará de forma indefinida previa aprobación de la Comisión de Expurgo en virtud de lo regulado en la Ley 16/1985, de 25 de junio, del Patrimonio Histórico Español y la normativa específica aplicable en su caso.

**¿Cómo protegemos la información?**

Como Administración Pública, aplicamos las medidas técnicas y organizativas que nos dicta el Esquema Nacional de Seguridad. Este contempla una serie de recomendaciones para tratar de garantizar la seguridad de los sistemas de información y así evitar el robo, alteración o accesos no autorizados a datos.

**¿Cómo puede ejercer los derechos regulados?**

Para poder mantener en todo momento el control sobre sus datos tienes derecho acceder a su información personal, así como a solicitar la rectificación de los datos inexactos o, en su caso, solicitar su cancelación o supresión. En determinadas circunstancias, y por motivos relacionados con su situación particular, podrá oponerse al tratamiento de sus datos. De igual forma, puede ejercer el derecho de limitación del tratamiento de su información personal, solicitándonos su conservación y también la portabilidad de sus datos.

El ejercicio de derechos es personal y por ello necesitamos identificarle de modo inequívoco. Puede ejercer sus derechos de dos modos:

▪ Mediante el envío de un mensaje de correo electrónico.

Para ello, utilice esta dirección: [dpd@usal.es](mailto:dpd@usal.es). Únicamente atenderemos las solicitudes que se realicen desde cuentas de correo electrónicos proporcionadas por la Universidad de Salamanca o que consten en nuestras bases de datos previa identificación de su titular.

▪ Mediante la presentación de un escrito en nuestro Registro o por correo postal dirigido a:

Secretaria General

Universidad de Salamanca

C/ Patio de las Escuelas Menores, nº 1

C.P. 37008, Salamanca.

Debe aportar la siguiente documentación acreditativa:

▪ Acreditación de la identidad del interesado mediante cualquier documento válido, como DNI o pasaporte.

▪ Nombre y apellidos del interesado o, cuando corresponda, de la persona que le represente, así como el documento acreditativo de tal representación.

▪ Petición en que se concreta la solicitud.

▪ Dirección a efectos de notificaciones, fecha y firma del solicitante.

▪ Documentos acreditativos de la petición que formulas, si corresponde.

▪ En caso de la rectificación o cancelación, indicación del dato a rectificar o cancelar y la causa que lo justifica.

**¿Quién garantiza los derechos? ¿Ante quién puede reclamar?**

En caso de que desee presentar una reclamación u obtener información adicional sobre la regulación del tratamiento de datos personales en España, la autoridad competente es la Agencia Española de Protección de Datos (Jorge Juan, 6 28001-Madrid).

Fdo._______________________________________
